# Biographical Sketch of the Late John Alexander Schetky

**Published:** 1825-09

**Authors:** 


### OBITUARY.

***Biographical Sketch of the late* John Alexander Schetky, *Deputy Inspector of Army Hospitals, Fellow of the Royal College of Surgeons of Edinburgh.***

This excellent and distinguished medical officer, whose premature death at Sierra Leone excited so deep and general feelings of regret and sympathy in his own department, and among his numerous friends, was born at Edinburgh, in March, 1785.

Mr. Schetky received his general and preliminary education at the High School and University of Edinburgh, and was early sent to cultivate drawing at the Trustees' Academy, under the late Mr. John Graham, of whose instructions he spoke with much gratitude; where he had the good fortune to have as fellow-students his distinguished countrymen Wilkie and Allan, with whom he ever afterwards maintained habits of the greatest intimacy and friendship.

Mr. Schetky commenced the study of medicine under the late Mr. Thomas Wood, Fellow of the Royal College of Surgeons of Edinburgh, with whom he served an apprenticeship. Having completed the usual course of medical study under the Professors in the University, and other teachers of medicine and surgery in Edinburgh, he obtained the diploma of surgeon from the Royal College of Surgeons early in 1804; and, in March of that year, entered the medical department of the army as temporary hospital-mate, attached to the third Dragoon Guards, then in Ireland.

After passing the requisite examination before the Medical Board in Dublin, he was appointed assistant surgeon to the same regiment, and served with it in Ireland and England until April 1809, when he embarked with the regiment for Portugal; but, from sickness, was reluctantly forced to return to England, on leave of absence, in the autumn.

With his characteristic zeal, Mr. Schetky rejoined his regiment in December, and continued on active service with the third Dragoon Guards until August, 1812, when he was promoted to the rank of surgeon to the Portuguese forces under Marshal Lord Beresford. During the whole of Mr. Schetky's services with the third Dragoon Guards, the active and unwearied discharge of his professional duties, the rectitude of his conduct, and the kindness of his disposition, procured for him the uniform confidence and support of his colonel, Sir Granby Calcraft, the warm friendship of his brother officers, and the respect and attachment of all ranks; a result which, happily, such qualities rarely fail to command in the British service.

In the Portuguese service, Mr. Schetky did the field duty of a brigade surgeon of the seventh division, commanded by the Earl of Dalhousie, in the different actions in which that division had a share, until the termination of the war in 1814. His meritorious conduct in this service obtained for Mr. Schetky the warmest approbation of the commander-in-chief, and of all those with whom he served; and when, in consequence of the peace, a reduction in the number of the British medical officers attached to the Portuguese service took place, he was selected by Marshal Beresford as one of those whom his lordship wished to retain in that service. Mr. Schetky declined his appointment, from a natural desire, after a long absence spent in unceasing activity and turmoil, to revisit his native land, to enjoy for a time the society of his aged father and affectionate family, and to renew those liberal studies, both general and professional, of which he ever continued to be so ardent a follower.

Mr. Schetky remained on half-pay from 1814 till September 1819; and, during this interval, engaged, with his usual zeal, in the study and practice of his profession in Edinburgh, and in the ardent cultivation of the arts of drawing and painting, to which he was deeply devoted.

During this period, Mr. Schetky revived many connexions which had been interrupted by his long absence, and formed many that were new; and no one could be more happy than he was in enjoying the esteem and respect of his medical brethren, as well as of all those whose acquaintance he formed in society. Among others, he renewed his intercourse with one of his earliest teachers and friends, Dr. Thomson, then professor of military surgery, who was among those whose friendship he prized most highly, and for whom he always expressed the warmest respect and attachment. When a student, he had made for Dr. Thomson several anatomical and pathological drawings; and now, in order to promote those objects in science which he found Dr. Thomson still zealously pursuing, he devoted his talents as a painter partly to the delineation of external and internal pathology. His drawings and paintings of these subjects are distinguished above most others of the same kind, not only by the accuracy and distinctness with which the particular objects to be shown are delineated, but also by their spirit of design, their originality in style, and excellence as works of art; but we know that Dr. Thomson now values those in his possession more peculiarly as memorials of one of his earliest pupils, for whom he felt a sincere affection and esteem, and in whose welfare he always took a deep interest.

At this time Mr. Schetky became a Fellow of the Royal College of Surgeons of Edinburgh; and, in conformity with the regulations of the College, published a Probationary Essay on Syphilis and Pseudo-Syphilis, which he inscribed to his friends Dr. Thomson and Dr. Maclagan, physician to the forces. Between Dr. Maclagan and Mr. Schetky an intimate connexion had subsisted since their boyhood. They had been fellow-students in the same schools of instruction and service, and had now returned to a home where they had many ties and objects in common, after having been long employed in the same department, and engaged in the same scenes. This friendship was only terminated by Mr. Schetky's premature death; in lamenting which, it is the anxious desire of the survivor to evince his respect for the memory of the companion of his youth, and the friend of his maturer years, by endeavouring in this slight, though sincere tribute, to record his excellence and his worth.

In September, 1819, Mr. Schetky was gazetted to full-pay, and employed at the general hospital at Fort Pitt, Chatham; where Sir James M'Grigor, by the establishment of a museum of morbid anatomy, had lately added another claim to the gratitude of his department, and to the respect of the medical profession. Mr. Schetky valued highly and justly the efforts of the head of the medical department of the army to promote the interests of medical science and the honour of that department, as well as the kind interest he took in the individuals composing it. While in Edinburgh, Mr. Schetky had occasionally sent delineations of such subjects as he considered valuable or interesting to the museum at Chatham; and now, while he zealously performed the duties of the surgical department, he applied his talents in drawing and painting to the illustration of different subjects in pathology which presented themselves in the hospital and in the preparations in the museum; and he was latterly occupied in finishing those lithographic engravings which form the fasciculus of Morbid Anatomy, lately published under the authority of the Medical Board, and dedicated to his Royal Highness the Commander-in-chief. This work must be regarded as a valuable addition to the illustrations of pathology; and, had this intelligent medical officer and accomplished artist lived, it cannot be doubted that, from the stores of this valuable museum, as well as from other sources, he would still more materially have enriched this important branch of medical science.

Mr. Schetky's services at Chatham were highly valued by Dr. James Forbes, the able medical chief of the hospital at this period, as well as by his other colleagues. They were no less highly appreciated by Sir James M'Grigor, who says, that to Mr. Schetky the museum at Chatham owes much indeed, and the fasciculus of engravings every thing; and has conjoined with this honourable testimony the warmest approbation of Mr. Schetky's general services, the expression of deep regret for his death, and of respect for his memory.

During this period of his service, Mr. Schetky was enabled to make a few visits to his home, to which his family and friends now look back with a melancholy satisfaction. But we mention these at present, in order to notice one of them as highly characteristic of the kindness and warmth of his disposition, and of the disinterested zeal by which he was always actuated. He had learnt that his immediate presence in Edinburgh might be of essential importance to the interests of a valued and attached friend; when, unsolicited, and regardless of the fatigue or of the serious inconvenience to which he knew this journey must subject him, he, after obtaining a short leave of absence, immediately set out for Edinburgh, where he had the satisfaction to arrive in time to assist his friend in obtaining the object he had in view. The surprise occasioned by his unexpected presence on this occasion, was only equalled by the pleasure and gratitude his friend experienced, and by the admiration he felt of so generous a proof of the friendship of one for whom he had the highest esteem and regard.

In August, 1823, Mr. Schetky was promoted to the rank of deputy inspector of hospitals, for service on the coast of Africa; an appointment which, prompted by an honourable ambition of advancement, an ardent spirit of inquiry and observation, and no unnatural confidence in the strength of a vigorous constitution, he had long been desirous to obtain.

He embarked for Sierra Leone in December, 1823, and arrived there on the 5th of February, 1824. He immediately devoted himself with great energy to the duties of his new station, which, by the removal from Sierra Leone of the rest of the medical staff, in consequence of the war with the Ashantees, had become doubly arduous. He was also, soon after his arrival, appointed a member of the colonial council, which involved additional occupation.

In all his letters he manifested the most lively sympathy in the state of this colony; of which, the various tribes, with their habits and customs, the richness and picturesque nature of the scenery, and the objects of natural history, of which he was forming a museum, afforded him the greatest interest and pleasure. During the time he was at Sierra Leone, nearly six months, the uniform good health he enjoyed seemed to justify his own confidence, and to encourage the hopes of his family and friends that he would be able to resist the baneful influence of the climate; but unhappily these too soon proved delusive.

In proceeding on official duty to Cape-Coast Castle, in August 1824, he was attacked, a few days after embarkation, by the fever of the country, which proved fatal on the 5th of September, the day the vessel arrived at Cape-Coast Castle.

Thus died, at an early age, in the full career of honour and usefulness, this excellent and amiable medical officer, deeply lamented in that colony to which his services had already given the promise of so much benefit, and by that department of which he was so valuable and respected a member; but leaving a still deeper blank in the hearts of a family worthy of such a brother, and in many a circle of friends, to whom his gentle and generous nature, sincerity, and singleheartedness, had deservedly endeared him.

Mr. Schetky's character was of no ordinary cast. He was remarkable for his early habits of study: even while a boy, reading and drawing, with an ardent and observant admiration of the beauties of natural scenery, constituting his chief amusement. In the midst of his various occupations, he cultivated painting with that enthusiasm for the art, and that powerful perception of the beauties and sublimities of nature, so essential to excellence, and which so strongly mark his numerous sketches and paintings of the scenery of the Peninsula and of his native country.

The writer of this notice is too imperfectly qualified to hazard the expression of his own opinions as to the particular character and merits of Mr. Schetky as a landscape-painter; but he is happily enabled to give those of a highly gifted individual, himself an acknowledged master both of the theory and practice of this delightful art, which will confer on this slight memorial a much higher value than it otherwise possesses.

Mr. Schetky had scarcely less enjoyment, and no mean knowledge, in the sister art of music, in which all his family have been singularly accomplished, and in whom it would seem to be an hereditary endowment.

To these Mr. Schetky added a love of literature, which his facility of acquiring languages enabled him the more readily to indulge,—an enthusiastic admiration of the efforts of genius in every department of human excellence,—an ardent pursuit of all that was honourable and useful in his profession; and, though possessing a high relish for social enjoyment, he never permitted his private pleasures to interfere with the discharge of his public duties, or with the offices of humanity or friendship. He began life without the advantages of interest or connexion; but his talents and industry, joined with the integrity, active benevolence, kindly warmth, openness, and simplicity of his conduct, secured to him not only the deep affection of his family, and the warm attachment of his friends, but the confidence and support of his superiors in the army, and that professional character so honourable to his memory. His whole conduct was, indeed, a practical illustration of the purity of those moral and religious principles which he had imbibed in early life, and which he cherished to its latest period. In his affectionate correspondence with his family, he often dwells on the value and importance of these early principles with peculiar earnestness. They were his guide and support throughout the vicissitudes of life, and are now the best consolation of that family, who, by his untimely death, have been deprived of so invaluable a brother.

---

